# The breakthroughs of caregivers in a parent-implemented intervention for children with neurodevelopmental disorders in Hong Kong, China: an exploratory qualitative study of change mechanisms

**DOI:** 10.3389/fpsyt.2026.1754567

**Published:** 2026-02-18

**Authors:** Cecilia Hok Man Wong, Camille Kuen Yu Chan, Paul Wai Ching Wong

**Affiliations:** 1 Faculty of Social Sciences, The University of Hong Kong, HongKong, Hong Kong SAR, China; 2 Department of Social Work and Social Administration, Faulty of Social Sciences, TheUniversity of Hong Kong, Hong Kong, Hong Kong SAR, China

**Keywords:** autism (autism spectrum disorders), caregiver, change mechanism, environmental cue, neurodevelopemental disorders, parent-implemented early intervention program, self-care, theory of change (ToC)

## Abstract

**Introduction:**

Parent-implemented intervention (PII) trains caregivers to be the interventionists to offer tailored learning opportunities at home and is used to promote the positive development of children with neurodevelopmental disabilities. Its effectiveness hinges not only on learning and implementing the strategies, but also on the beliefs, wellbeing and capacities of the caregivers. The existing literature focused on the acquisition and application of skills, yet little is known about the inner experiences and transformation of caregivers. This study explored their changing processes and focused especially on their breakthroughs.

**Methods:**

Twenty-two caregivers participated in five semi-structured focus groups in Hong Kong, China. They had all completed the localized version of the World Health Organization Caregiver Skills Training. Reflexive thematic analysis was adopted to analyze the qualitative data and construct codes and themes.

**Results:**

There were three major breakthroughs which caregivers experienced leading to positive outcomes for themselves and for their children. The first one was the acquisition of self-care practices that helped caregivers recognize their own physical and emotional exhaustion and cultivate capacities for childcare. The second was on environmental settings, in which the caregivers learnt to adjust the home setup to promote parent-child engagement and the building of play routines. The third was on developing the discernment of the window to intervene. Caregivers learnt to adjust their pace to align with their children’s, follow into the children’s world and cultivate empathy towards the young. Being able to discern when and how to create teaching opportunities allowed caregivers to be more relaxed, and children enjoyed interacting with their caregivers more.

**Conclusions:**

This study offers insights into the theory of change of PII by unveiling the transformation processes of caregivers and identified the key factors promoting positive outcomes. Through focusing on breakthroughs, it also sheds light on the inner struggles and rewards the parents experienced as the primary caregivers and interventionists. It invites clinical professionals to consider the wellbeing of caregivers as one of the key social determinants of the success of PIIs, and explore how self-care, environmental setup, and discernment to intervene shape immediate and long-term clinical outcomes.

## 1 Introduction

Neurodevelopmental disorder is a group of disabilities characterized by disruption to brain development and has its common onset in childhood (1, 2). Globally, the prevalence rates stood at: intellectual disability 0.63%, attention-deficit/hyperactivity disorder 5-11%, autism spectrum disorder (ASD) 0.7-3%, specific learning disorder 3-10%, communication disorders 1-3.42%, and motor disorders 0.76-17% (3). For children facing such challenges, a comprehensive early intervention is crucial for promoting their lifelong development as it makes use of the window of brain plasticity at an early age for maximum learning (4).

Parent-implemented intervention (PII) (or parent-mediated intervention) is an effective early intervention for children with neurodevelopmental disorders. It employs a triadic model which requires the caregivers to first learn the strategies from clinical professionals, then offer training to their children at home (5). It is not designed to replace but complement the clinician-implemented treatment so that children can have more tailored learning opportunities (6, 7). Research has shown that PIIs are useful in promoting children’s adaptive behaviors, social communication skills, motor skills, cognitive skills and learning abilities while reducing maladaptive behaviors (8–10). With caregivers being equipped with the specialized knowledge and skills to care for their children, they too benefit from PIIs. Being empowered, their parental stress is alleviated, and their confidence is boosted (11, 12). PIIs can also create positive impacts on families when other caregivers are willing to learn from the trained-caregivers and put the new skills into practice (13, 14). Hence, PIIs have strategic values of strengthening the functioning of the children, parents as well as families. Scientific evidence reveals that although gene abnormalities and congenital brain lesions contribute to neurodevelopmental problems, environment and experience also play a role in shaping the brain development thus altering its trajectories (15). PIIs are therefore uniquely powerful as they nurture the natural family environment which is conducive to healthy child development (16).

With the effectiveness of PIIs being validated, more qualitative studies were conducted in recent decades to explore why and how PIIs worked. These studies were on three domains: (1) Documenting the outcomes of PIIs, (2) identifying barriers and facilitators, and (3) investigating the change processes in the interventions. On the examination of outcomes, the existing qualitative research has found that PIIs are useful in helping the caregivers to make sense of and accept their children’s atypical development, equip them with new parenting strategies, strengthen parent-child relationship, promote children’s development, and support family members to acquire new caring skills (17–19). On the barriers and facilitators, research has showed that program design (e.g. the pace of learning, the possibility of integrating the skills into daily routines, and the match between the program and the participants), trainers’ competencies and relationships with the parents, parents’ commitment and self-efficacy, and logistics (e.g. time, location, and availability of childcare) are factors affecting caregivers’ participation in the PII training and their application of skills at home (17, 19–22). On the change processes, research has shared a similar three-step process: (1) The caregivers are empowered and experienced an increase in self-efficacy, (2) they practice applying the skills, then (3) they witness the changes in their children (23–25).

While the existing qualitative studies have provided rich accounts of the lived experiences of PII participants, we still know little about the internal changes of caregivers. For example, Holtrop et al. set out to examine the process of change, and they identified three steps: Attempt, Appraise, and Apply. These steps described the actions the parents took to apply their learned knowledge and skills, yet this action-oriented process did not unveil the inner experiences of caregivers (23). Mejia et al. sought to examine the changing mechanisms of PIIs (24). They identified three types of changes—psychological mechanisms behind changes, behavioral changes in the parent, and changes in the children—and weaved them into a change model with hypothetical causal relationships. This work drew new light to the psychological mechanism of caregivers; however, as psychological changes were not the only focus of this study, the inner experiences they captured were limited to self-efficacy and emotional regulation. Frost and Ingersoll purported to delineate the theory of change, and they identified different pathways describing how parents reacted to different ingredients of the intervention (25). An example of these pathways was “Collaborative goal setting > Parent empowerment, self-efficacy, and motivation > Caregiver practice outside of session > Child skill development”. As explained by the authors, the goal of this study was to find out how learning and motivation supported the caregivers’ implementation of skills. Hence, it was designed to investigate how motivational factors elicited desirable actions rather than openly exploring the inner changes of PII participants. Thus far, the literature has only painted a broad stroke description that parents are likely to experience an increase in motivation, confidence and skills, and there has not yet been a study dedicated to following into the caregivers’ inner experiences of changes.

In this research, we employed the qualitative research approach to explore the lived experiences of caregivers in PIIs. We asked our caregiver respondents to describe the changes in themselves, their children and their families, and we focused the discussion particularly on their breakthroughs that propelled positive outcomes. This emphasis is important because PII, as an intervention that has adopted an experiential approach to adult learning, requires caregivers to move beyond grasping the skills and concepts to experiencing transformation through active experimentation and reflective observation (26).

Breakthrough also marks a time of paradigm shift when caregivers overcome their obstacles, allow a new belief to take root or chart a new course of actions. Underscoring breakthroughs opens the doors for us to apprehend their concerns, struggles and needs. In PIIs, the caregiver-trainees are the main interventionists as well as the primary caregivers who have their personal and familial responsibilities, expectations and challenges. The effectiveness of PIIs is therefore determined not only by the competencies in learning and applying of skills, but also the capacities, beliefs, habits and wellbeing of caregivers. Through discussing how they let go of their old beliefs and practices to adopt the new ones, we join the caregivers into their stories to apprehend the dilemmas they faced and identify factors affecting their acquisition and implementation of skills. The insights on the change mechanisms will help inform the theory of change in PIIs.

When the existing literature gauged the success of PIIs mainly through a methodological lens—the adherence to protocol, the quality of intervention delivery and the level of dosage, this research sheds light on how the capacities, beliefs and wellbeing of caregivers shape the processes and outcomes of the interventions (5).

As caregivers’ experiences are of concern, we need to situate our understanding in the cultural context of Hong Kong, China to make sense of the local experiences. Previous research has showed that although Hong Kong has a relatively well-developed medical system, the caregivers of children with neurodevelopmental disorders are still encountering many challenges in help seeking. The private consultations are expensive and there are no standardized intervention models (27). The public services are constantly under the pressure of overwhelming demands and the lack of physical doctors and civil service staff, some children need to wait for a few months to over a year for their assessments at the Child Assessment Centers of the Department of Health (28–30).

The household sizes are generally small in Hong Kong with over 90% of them had 4 persons or less in 2021 (31). In this living environment, the caregiving responsibility sometimes fall mainly on one caregiver, while some have more supportive spouses and grandparents to share the tasks (14). Employing domestic helpers to support household management and childcare is common as around one-third of the households with children aged 12 or below were hiring foreign domestic helpers in 2021 (32).

Aside from the challenges in daily caregiving, the caregivers are also facing tremendous stress from different fronts. They worry for their children’s academic performance and future development, some of them are ashamed to share the news of their child’s diagnosis with other family members, some blame themselves for their children’s disability, and many have experienced stigma from the society (33–35).

## 2 Methods

### 2.1 The intervention

The World Health Organization Caregiver Skills Training (WHO-CST) was introduced in Hong Kong, China in 2018 through the Family Support Team of the Jockey Club Autism Support Network program (JC A-Connect) at the University of Hong Kong (36–38). This course was a PII that targeted at caregivers of children aged 2-9 with developmental delays or disabilities. It aimed to equip them with skills to promote their children’s engagement, communication, positive behaviors and daily living skills, while strengthening caregivers’ confidence, psychological wellbeing and connection with their children. This intervention contained 9 group sessions and 3 individual home coaching sessions. After the initial adaptation, the local master trainers further localized the course to respond to the training and learning needs of the professionals and caregivers in Hong Kong in 2021. The PII studied in this research was this localized version. In Hong Kong, this PII was mostly open to caregivers with children aged 2-6 who were suspected or confirmed to have ASD.

### 2.2 Research design

We conducted an evaluation study in 2023 and 2024 to examine the impacts and sustainability of the localized WHO-CST in Hong Kong, and this study was part of this evaluation research. The research was approved by the Human Research Ethics Committee of the University of Hong Kong with a reference number of EA240065. Two articles had been published from the larger research, they were to report the intervention outcomes (14, 39). Qualitative method was adopted to investigate the lived experiences of the caregiver-participants. When quantitative study is limited by the predetermined variables, qualitative approach offers room to explore the unknown territories. This study was grounded in social constructionism and social interactionism (40). We were aware that the experiences shared by our research respondents were constructed subjectively by their sense-making and their own interpretations that were influenced by a wide range of factors such as social culture and values. Thus, having multiple accounts of stories offered us an array of perspectives that enriched our understanding. Social interactionism drew our attention to the power of social exchanges in shaping people’s understanding. We recognized that the lived experiences were shaped by the interactions during the interventions, after the interventions and even in the data collection sessions.

### 2.3 Participants

We recruited the caregiver-participants who had completed the localized WHO-CST at the time of data collection. Target-convenience sampling was used as the main method. Invitations and information sheet were sent through the organizations that offered the PII to their graduates, caregivers who were interested in participating in the research could complete a brief online survey to provide the research team their contact methods and availability. Our researchers contacted each interested party through phone to introduce the research and arrange them into focus groups. We also used the snowballing method for recruitment by asking the participants to refer their friends and families who had also completed the PII.

Twenty-two caregiver-participants participated in this research. Twenty-one of them were female, and one man was the husband of one of our respondents. They were mothers (18), father (1), grandmother (1), auntie (1), and foster mother (1). Sixteen of them were in a marriage, three were singles, and three had separated with their partners. Two attained a master’s qualification or above, seven with a bachelor’s degree, and thirteen had completed secondary school or post-secondary diploma. Fourteen were homemakers, five were in a full-time job, two took up multiple jobs, and one worked in a part-time capacity.

### 2.4 Data collection

Five focus groups were conducted, and each lasted for about 1 to 1.5 hours. Three groups were conducted virtually through ZOOM, and two were physical meet-up at the University of Hong Kong and at a social services center. Participants were asked to read an online information sheet, complete a consent form and a short profiling questionnaire prior to the focus groups; all of them consented to participate voluntarily. The discussion was semi-structured, participants were asked to share about their experiences in the PII and the changes they observed in themselves, their children and other family members. To further focus them to their breakthroughs, we asked, “What were the points which you found yourselves being “unlocked”? (“Unlock” is a local expression meaning that we finally let go of what previously limiting us and can now unleash our potentials.) What motivated these breakthroughs?” The dynamics of the discussion varied across groups. The participants were generally more responsive to the familiar faces (their peer-trainees in the same PII group), but they were mostly comfortable to share their struggles and concerns in the caregiver focus groups. Given that this PII targeted mainly at Cantonese speaking Chinese in Hong Kong, this was also the profile of our respondents. As an incentive, all respondents received a supermarket voucher costing HKD100 and a set of JC A-Connect souvenirs after the data collection sessions.

### 2.5 Data analysis

All discussion was recorded and transcribed, names were replaced by participant codes in the transcripts, and all data were stored securely to ensure confidentiality. For data analysis, we opted for reflexive thematic analysis for it encouraged us to engage deeply with the data and allowed us to immerse in the meaning creation process to generate informative themes and frameworks for the advancement of the field (41, 42). Choosing this approach, we recognized that our interpretations of data and our generation of findings were influenced by our own values and training. Our research team consisted of a clinical psychologist (PW), a counselor-trained evaluation researcher (CW), and a researcher (CC) with experiences in providing mental health training to clinical professionals. We did not know the respondents in advance and had no previous interactions with them other than the contacts for arranging the focus groups. With our different background, PW was strong in assessing the clinical conditions of the clients and identifying factors affecting the intervention outcomes. CW was keen in finding out patterns, anomalies and contextual factors that could shape the respondents’ perspectives, preferences and behaviors. CC was sensitive to the respondents’ wellbeing, stories of changes, and interactions among respondents. When we brought ourselves into the data analysis, we found that our differences helped widen our focus and deepen our understanding. As an exploratory study, we followed into the respondents’ stories and applied the inductive approach to generate codes. The qualitative analysis software, NVivo, was used for engaging data and processing the codes.

We followed the suggested procedures of conducting a reflexive thematic analysis and proceeded gradually from familiarization to code generation then theme construction (41). We started with researchers reading the data separately to generate provisional ideas before sharing our initial notes and impressions with each other. Next, we individually applied open coding to code the same set of data and met to share and discuss our lists. The initial coding exercise was to generate new codes to describe the conditions and characteristics of the respondents, their experiences in the training and applications of skills as well as the changes in themselves, their children and family members. Examples of coding were “emotional exhaustion from prolonged child caring responsibility and neglection of own needs”, “develop play routines by setting up specific table and chairs for playtime”, and “trainers commented that caregiver was speaking too fast and too much”.

We then returned to our data to further review and refine our codes before constructing our themes. Giving researchers time to engage with the data individually allowed us room to mobilize our different expertise to make sense of the data and develop our own informed interpretations. In these later rounds of coding exercises, our research team reviewed the codes by checking their validity in the specific context in the data and adding more substances to them by comparing similar codes across participants and focus groups. Examples of our considerations were “How did different respondents experience emotional exhaustion? What contributed to their emotional exhaustion?” “Was setting a chair and table a common strategy? Why would it work for the families that adopted this strategy?” Through iterating this investigation process, we sought to interpret the data by putting them into the context, comparing the different manifestations and linking the ideas. Themes were generated, reviewed and revised together.

Throughout the analysis, we needed to mobilize our creative thinking and expertise to decide what constituted a breakthrough, how we made sense of the change mechanisms, and how to create themes that were informative and easy to grasp and apply. Heeding the suggestion from Braun et al., we did not aim at achieving data saturation for we believed that we could always learn more and interpret more with new data (43).

## 3 Results

From interacting with the data, we generated three overarching themes describing the breakthroughs the caregivers experienced which propelled positive changes in themselves and in their children, they were: (1) Self-care for the wellbeing of self and the child, (2) home setup for parent-child joint engagement and routine building, and (3) discernment of the window to intervene. Each overarching theme contained two to three subthemes.

### 3.1 Theme 1: self-care for the wellbeing of self and the child

Caregivers joined the PII expecting to acquire skills for caring for their children; many were surprised to learn about the importance and practice of self-care. They described this learning transformative as it laid a strong foundation to sustain their caregiving journeys.

#### 3.1.1 Attending to one’s own needs to cultivate strengths and capacities for childcare

Most caregivers who recognized the values of self-care described their transformation in a similar approach: Before the PII, they were very consumed by the needs of their children. They tended to invest all their emotional and physical energy on their children; hence they neglected their own wellbeing. In the training, the caregivers were guided to attend to their own thoughts and feelings, and they began to feel their own exhaustion. Through reflections, they realized that they needed to first take care of themselves so that they would have the strengths and capacities to care for their children. A respondent employed a tree analogy to illustrate her view.

“It is good that the course taught parents to manage their own emotions. Many times, we focused entirely on our children thinking that they were the ones who had problems, and we dedicated all our attention on teaching them. We were not aware of our own emotions, focused too much on the child and neglected ourselves. Like a tree, you need to be strong so that you have plenty of leaves to provide shades for others. If you keep consuming yourself and become barren, how can you protect your child?” (C12, a mother).

Some participants added that they had to attend to their own emotional needs as well as physical needs so that they could be physically strong to take care of their children, especially when the children were sick.

“I didn’t think about taking care of myself so that I would have the strength to care for others. After this course, I changed my mind … At that time, Covid and flu were around, and my child just began schooling. In many occasions, when children fell ill, they spread the germs to their mothers. This was a time when I had to take good care of myself so that I could care for my child.” (C10, a mother).

Self-care was particularly important for caregivers of children with special needs as these children were more vulnerable and required much care, explained by a respondent.

“I agree that we have to first take care of ourselves so that we can care for our children. If we fall down, our children who have ASD or other special needs will not be able to care for themselves.” (C13, a mother).

#### 3.1.2 Recognizing own influences on children’s wellbeing

Self-care, as perceived by some participants, was also for the wellbeing of the children as caregivers’ emotions had direct influences on their children’s. A mother linked her own happiness to her child. In our researchers’ view, the happiness of a caregiver would open a door for their children to experience happiness.

“It is tough to be a parent of a child with special educational needs. Sometimes we forget our own needs and rush to sacrifice everything for our children. What I find precious of this course is that it reminds us to care about our own emotions, because when you are happy, your child can then be happy. Your feelings directly impact your child.” (C9, a mother).

While positive emotions in one could ignite the positive emotions in others, negative emotions were contagious, too. More than half of the respondents recalled how their negative feelings, expressions and actions kindled negative emotions in their children and caused a vicious cycle of emotional outbursts among them. Whereas some of them shared the stories of temporary defeats in which they were overtaken by their anger and frustration, some also shared their stories of victories.

“This course talked about meditation. The trainer led us into the meditation exercises. Sometimes, I would practice on my own. Before the course, when my son had a heightened emotion, I would be frustrated and had an emotional outburst. This further stimulated my son’s emotions. After learning meditation in the course, I know how to calm my own emotions. Even when my son is very frustrated, I would remain calm to discuss with him, he will listen. If his emotions are too strong or he wants to cool down, I may hug him. Hugging him is important, too. We remain silent, hugging him makes us feel peaceful.” (C11, a mother).

Taking a break (time-off) to allow all parties to calm down was a common strategy shared among the respondents. A father shared that he and his wife became more aware of their own emotions. When they found themselves getting angry, they would seek help from their partners.

“Sometimes we would find ourselves at the red or yellow light, then we would take a break. Let’s say when I am at the yellow light and am not in my best condition, I would say to my wife: You take care of him! (When I am irritated), I tend to scold him and be mad at him, so I stop teaching my son at that time. My wife does the same (when she is irritated).” (C21, a father).

Some caregivers also taught their children to recognize different emotions. A caregiver shared that her daughter could recognize the anger in herself and in her mother and pointed it out.

“Now when she is angry, she will say, “mom, you are angry.” I then ask if she is angry, too? When both of us recognize our anger, I will say, “let’s take a five-minute break before resuming what we are doing. This is much better than keep arguing.” (C10, a mother).

One caregiver explained that as adults, we could find comfort from people and things when we were upset. However, for children, their parents constituted a large part of their worlds, hence they could find limited or no buffer to counteract the negativity from their parents. Therefore, the impacts parents have on their children were much stronger than what we experienced as adults. To help the children to build a sense of security, the caregivers realized that they needed to manage their emotions well so that they could remain emotionally stable and offer a safe haven for the young.

“Only when your emotions are stable then you child’s emotions can be stable. In our worlds, we have many things—families, intimate relationship, careers and friends—that would contribute to our emotions. However, in the children’s worlds, even they have friends, the parents would have taken up eighty percent of their time, or even their whole worlds are around their parents. The emotions that the parents offered are all that affect their emotions. When you have emotional stability, your child can then have emotional stability. When you are emotionally unstable, your children won’t understand why you are mad, and they would feel unsafe. Without this stability, how can they learn other things? Especially when our children are comparatively sensitive and have less sense of security; if the parents cannot offer them safety, they will feel like a stranger to this world.” (C12, a mother).

Other respondents also agreed that emotional calmness resulting from self-care was essential for promoting the children’s immediate and long-term emotional wellbeing.

#### 3.1.3 Constructing new positive self-image

Most caregivers cherished self-care as it allowed them to do a better job in childcare, a few owned it for themselves and expressed their joy in building a new self-image. They shared that the identity as a parent had overshadowed their own and they had failed to recognize their own values. There was a sense of hopelessness and helplessness. Nevertheless, when they practiced self-care, especially meditation, they gradually saw that they were as valuable as their children.

“I am glad that the course reminds parents to praise and love ourselves and to reserve time for meditation. I find that meditation makes a big difference. When we have to handle everything and feel unsettled, we fail to see a bigger picture and forget to cherish ourselves. We are not just parents, not just tools to care for our children, we are human beings.” (C15, a mother).

For some, praising one’s own efforts and celebrating for small successes in skills application became their new self-care practice. They kept practicing these after their training and used them to reinforce their positive identity. A mother demonstrated this self-appreciation at the focus group.

“When I applied this skill, I found that communication with my child became a lot easier. I can do it, and I appreciate that I can. I appreciate that I spent time to learn, and I sat around like this on Saturdays to learn the skills.” (C19, a mother).

### 3.2 Theme 2: home setup for parent-child joint-engagement and routine building

Interestingly, another breakthrough that resonated with the participants was changing their home setup. In the course, the caregivers were taught to set up a dedicated space for parent-child play sessions. For those families who did not have a small table and small chairs at home for their children before the PII, they reported that the new physical arrangement sparked instant improvement in their parent-child interactions and in their building of play routines.

#### 3.2.1 Environmental setup for parent-child joint engagement

Some caregivers shared that they have never considered the influences of environment on their children’s learning before the training. In the course, they learnt that they needed to have a decluttered space for the children to focus, and the seating arrangement ought to be comfortable for both adult and child to prolong the parent-child interactions. Also, the caregiver needed to come to the child’s eye level to encourage engagement and to facilitate the child’s reading of caregiver’s facial expressions and mouth movement when pronouncing a word.

Before rearranging the environment, caregivers found it difficult to engage their children. The children seldom looked at the caregivers during interactions, and they would walk away quickly. After learning how environment would influence parent-child engagement, the caregivers began to pay attention to the children’s comfort level and engagement level, and they adjusted accordingly. As the children grew up, the caregivers continued to adjust the home setup to accommodate the height and preferences of their children.

“I think environmental setup is very important … the child is now four years old. From two years old until now, we have kept adjusting the seating arrangement. As parents, we keep trying different setups to make the child comfortable to play. When he was younger and was unable to sit still, we let him sit in the higher chair at the adult table. Now when he can remain at his seat without walking away all the time, he sits in his own chair and parents sit on the floor. Our eye level needs to match with the child’s. Now that he is older and has his own chair, we need to find a seating arrangement that allows us to play with him and is comfortable for parents as well.” (C5, a mother).

Some caregivers reported that they did not think of buying children furniture before the PII assuming the children could share adults’ furniture at home like what they had done when they were young. Now as they were trained to observe how environment affected their parent-child interactions, they added a small table and chairs for the children to match with their heights.

“Before the class, I would not prepare a small table and chairs. When I was young, I thought I had a table at home, so I naturally used it for doing homework and writing. If we wanted to play, we would go to the playmat. The trainer taught us that you needed to be at the eye level of the children so that they would feel closer to you. After the class, my home has a new small table and two small chairs of the same height.” (C13, a mother).

#### 3.2.2 Environmental setup as an anchor for routine and relationship building

Not many caregivers were used to play with their children before the PII. In their training, some realized that they had not spent enough quality time interacting with their children as sometimes they would be occupied by their phones. Setting up a dedicated space for parent-child interactions served as an anchor to remind both parties of their playtime. A few caregivers observed that their children had associated the table and small chairs with playtime with caregivers. Over time, the children conceived an impression that it was fun to play with the caregivers and became interested in interacting with them. When the adults approached the small furniture, the children would happily join in.

“One advantage (of home setup) was that the child begins to form an association between the table and playtime. Even now, we may not have separate sets of toys, we could take turn to play with the same toy … With the learning from the class, the child now accepts taking turns. In his mind, I can play with my mom at the table, and playing with mom is fun.” (C9, a mother).

Two caregivers shared that the joint engagement developed in the play routines at the table helped the children to get used to interacting and learning from the parents, and this opened the children up for more learning opportunities in other settings.

“We need to be practical about how to transform. I think the transformation is not just around the playtime at the table or other playtime. You do not only play with your child, but you also support their doing of homework, other training and going out. These are also excellent opportunities for the child to learn. When you can synchronize with your child, you can help him to communicate with the world and promote his learning.” (C12, a mother).

### 3.3 Theme 3: discernment of the window to intervene

In the focus groups, when the caregivers reflected on their learning and implementation of skills, they highlighted how learning when to intervene became their breakthrough points. Through the guidance from their trainers, they became aware that they could not force the learning into their children, but they needed to align with their children’s pace and follow into their worlds in order to find opportunities to teach new skills. When they practiced a child-centered parenting approach, they developed more empathy towards their children.

#### 3.3.1 Aligning with the child’s pace

The trainers would sometimes provide feedback on the caregivers’ videos of playtime with their children. Many caregivers reported that their trainers urged them to slow down their pace of speaking and teaching. With the advice from the trainers, the caregivers started paying attention to their own parenting styles and the impacts on their children. They found that when they were speaking fast and speaking a lot, their children would be confused about their messages, and the efforts were in vain. With practice, they learnt to slow down to align with their children’s pace.

Being fast was not uncommon in this fast-paced city. A caregiver explained that it was the busyness and stress at work that drove them to speed up. Also, she shared that sometimes she would unconsciously compare her child with those of typical development, and this also propelled her to give a lot of instructions.

“I think I have slowed down. Probably because I was very busy at work and the work pressure was tremendous, so I tended to speak faster than others. In this course, you learn to adjust your pace to align with your child’s … Human beings tend to compare, to compare with the children of your friends. You see that that mom has spoken a lot, and her child perfectly understands her messages. However, I know where my child is at. Even I have completed the course for two years, sometimes I would make the same mistake of speaking too fast and too much. Then I will remind myself: My child can only absorb this much. I can save my breath and speak less.” (C3, a mother).

“In the beginning, we as adults thought that I had to keep teaching her, but you didn’t consider that when you used a full sentence or described the whole event, she basically could not decipher what you were talking about. Let’s say if your sentence has ten words, she will assume that you are only talking about the first word … So, teach her less, let her build a foundation. It will be easier for her to learn more.” (C20, a mother).

Other than being fast, the trainers also urged the caregivers not to ask a lot of questions.

“I learn not to pose many questions and not to give a lot of instructions. I need to give him room to think. Perhaps I can use a question to guide him to think about how he wants to play.” (C11, a mother).

“Trainer taught me some skills and asked us not to keep raising questions. She kept reminding us not to ask questions but to let our children play in their own ways. This made the children think that it was fun to play, and they would then be willing to play with us and build relationships with us.” (C10, a mother).

Through slowing down, the caregivers came to realize the importance of aligning with the child’s pace and communicating in ways which the children could understand. They learnt that when they wanted to support their children in acquiring new vocabulary, they needed to design their teaching based on the children’s current level.

“You just asked us which skill was the most useful. I think of one, but I have forgotten its name. If the child always wants certain thing, you can encourage him to name the thing. Gradually, the child will build up his vocabulary. One word becomes two words. Two words become three words. This skill is very helpful. Many of his vocabularies were built slowly, one word, then add one more. Now he is speaking more and more, thank to this skill.” (C21, a father).

#### 3.3.2 Following into the child’s world

Many caregivers reflected that the major breakthroughs in their parent-child engagement happened when they learnt to engage the children at where they were. They reported that before the PII, they would be angry with their children from time to time because they were not willing to follow the adults’ instructions, then the two parties would compete for the control over the game. In the course, they were trained to observe their children’s responses and preferences and try to join in the children’s play. Through practice, they developed more understanding of their children’s playing styles and patterns and became more attuned to mirroring their children’s actions. They were surprised to find that by refraining from taking the control, they could discover and create more windows to help the children to learn.

“The most useful skills would be waiting, observing and listening, then joining into the children’s play. We need to start from where the children are at … The course is divided the process into steps to help us apply.” (C20, a mother).

“I learn that I need to observe the preferences and interests of my child, see how he plays, follows his play method and imitate. When he looks at you, you can teach him some phrases or one word, you can succeed. It is no longer like the time when we were young: ‘No, the car has to be played in this way.’ ‘Those blocks have to be stacked like this.’ ‘You cannot wear like this. You need to follow mom. You can’t do this.’ This course teaches us that if he wants to stack the blocks in this way, just let him stack, then follow his preference.” (C19, a mother).

“About playing, I have some reflections. Why do you need to be so nervous during playtime? It’s not an exam, and it’s not about studying. Even when you are preparing for an exam, the more you force him, the poorer the outcomes will be. This applies more so to playing. He does not hurt himself, he does not hurt others, so why so nervous and demanding? He can play in his own ways. Two cars can go out at the same time.” (C1, a mother).

A few caregivers found that following into the child’s play indeed made the playtime easier for both. A mother shared that she was happy to discover that her child could learn even from repeating the same game, as she could add small techniques at each new round. She felt more relaxed when she realized that she did not need to look for many different games to demonstrate different techniques, but one would do the work. This new understanding opened her eyes to create more small opportunities to teach new skills.

“I want to share about “restarting the same game”. My impression was that I could not play with my son, even I worked very hard. I was using the adults’ perspectives to play with him. In fact, repeating the same game is good enough. Those times are important for them. I find that the pace does not need to so fast, being slow is good enough. Be repetitive and add new techniques. Those are the long struggling moments, but I find myself feeling more relaxed.” (C5, a mother).

#### 3.3.3 Parenting with empathy

Learning to align with the child’s pace and following the child’s play method helped the caregivers cultivate more empathy towards their children. They realized that young children were like adults who felt and thought in similar ways. To make sense of the child’s frustration, caregivers needed to think in their shoes.

“Even he is young, he also has feelings. You cannot think for them and plan for them just because they are young. This is the misunderstanding of most parents.” (C1, a mother).

“Indeed, putting myself into his perspective, many techniques are built upon empathy. It’s the same for adults. You have something, then I suddenly take it away from you. We wouldn’t appreciate it. We didn’t think that children would feel the same as adults. This skill is useful, empathy is important.” (C21, a father).

A mother shared that she had two children. The first one was of typical development, but the second had special educational needs. She applied the same parenting approach to the second child and was disappointed to find out he could not follow her instructions, she was frustrated. After she learnt about developmental delays and disabilities in this course, she was able to see from his son’s perspective and realized his helplessness. This changed her way of teaching her son.

“Joining this course helped me to understand more about children with special educational needs, and I lower my expectations. I have two children. The first one is of typical development. In many occasions, I wanted to use the same parenting method which worked for the first one, and I was disappointed that he (the younger one) failed to do what was told. ‘Why can’t you do this? You brother can, why can’t you?’ I was perplexed and frustrated. I wasn’t aware that I was rude to him and might scold him loudly. From the child’s perspective, he felt wronged. ‘What? I’m not getting it. This is it. Why is mom treating me like this?’ After joining this course, I learnt that he was using his own ways to play, and this understanding has helped improve our relationships … I can calm down; I can’t treat him like this … Now the child has a lot of improvements. I’m happier, my child is also happier. Our parent-child relationship is so much better.” (C7, a mother).

Some caregivers also shared that now they would take time to reflect on their interactions with the children to see if their expressions might have hurt them.

“Change? Just as this mom has shared, we would frequently reflect on our behaviors. Did I make a mistake? Was it not good enough? When I said those words, would I hurt him? The younger children might not show (their feelings).” (C2, an auntie).

**Figure 1:**
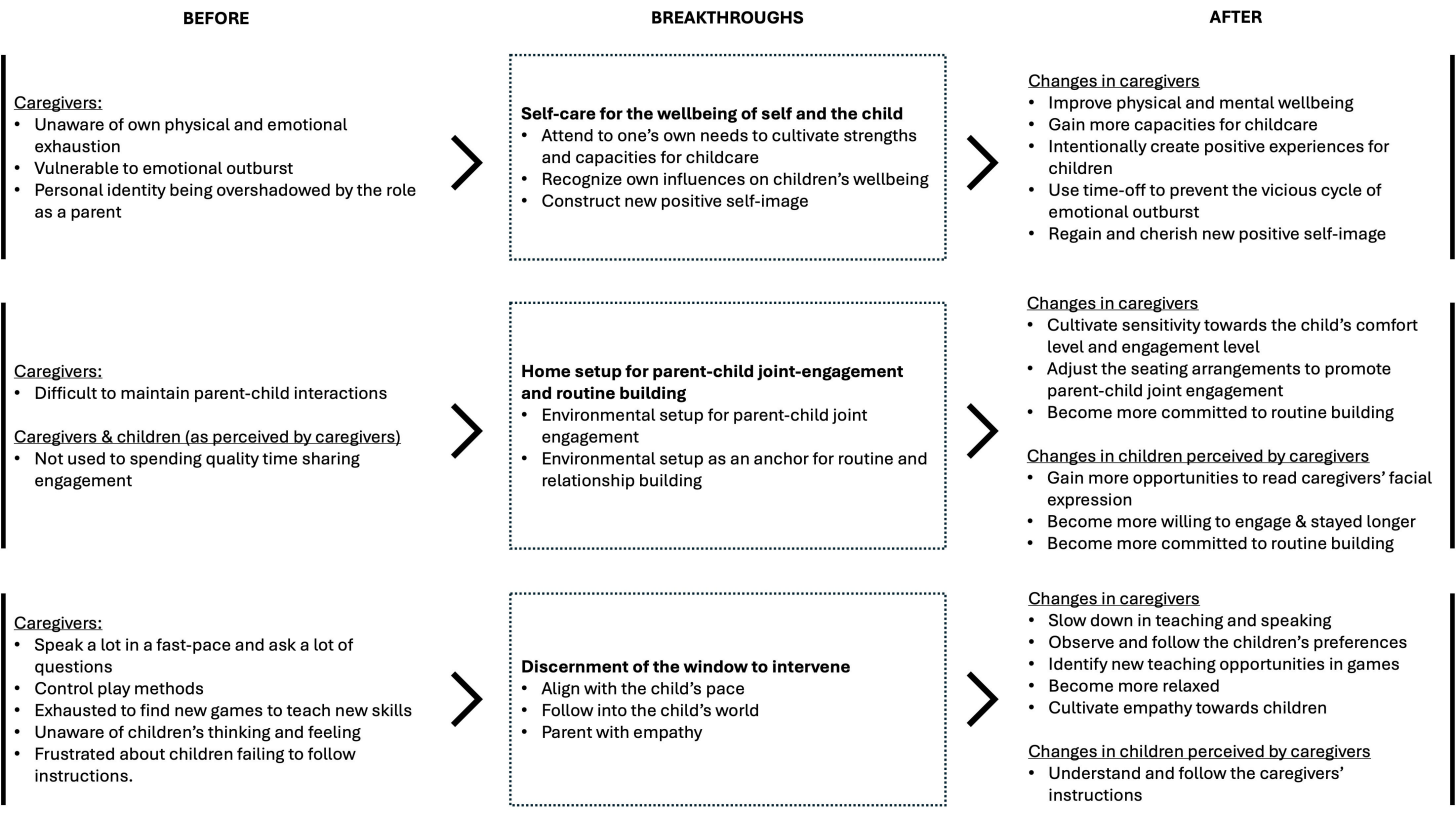
Summaries of the transformation of caregivers in the PII in flowcharts.

**Figure 1** has summaried the transformation of caregivers in a flowchart.

## 4 Discussion

This qualitative study aims to explore the transformation processes of caregivers in the PIIs, and it focuses especially on their breakthroughs. From the sharing of the caregiver-participants in the focus groups, we found three major changes: (1) Self-care for the wellbeing of self and the child, (2) home set-up for parent-child joint engagement and routine building, and (3) discernment of the window to intervene. As reflected by the respondents, these breakthroughs addressed their inner hurdles and helped create positive outcomes for the caregivers and their children.

The caregivers participated in the PII with the hope to acquire some strategies to care for and promote the development of their children who were experiencing developmental delays and disabilities. Many of them were suspected or diagnosed with ASD. To their surprise, the caregivers found that the course was beyond their expectations as they also learnt about self-care. Many caregivers reported that they were used to be completely occupied by the needs of their children, and they were unaware of the exhaustion that they were experiencing. Unlike the original WHO-CST, this localized course emphasized more on self-care with trainers teaching and practicing meditation with the caregivers in the group sessions. The caregiver-respondents found this teaching very helpful. Through paying attention to and getting in touch with their own emotions and physical conditions, they became aware of their own stress, the depletion they were feeling, and their suppressed personal identity. This realization shed light on their own psychological needs, and they became aware of the necessity of taking good care of themselves. They discovered that through attending to their own needs, they were indeed cultivating strengths and capacities to care for their children.

When the caregivers attended to their own emotions, they also became more attuned to the emotional fluctuations in themselves and in their children. With this new lens, they recognized the influences of their own emotions on their children and learnt that both positive and negative emotions were contagious. The insights motivated caregivers to create more positive experiences for their children and prevent themselves from imminent emotional outbursts. Although they were not able to hold their frustration in place every time, they found that they had gradual improvement in their emotion management and they could seek help from other adults when their negative emotions were heightened. In practicing self-care, some caregivers also reclaimed their own personal identities and constructed a new positive self-image.

Self-care was a breakthrough shared among the caregiver-respondents as this met their desperate needs. Caring for a child with special needs was already a difficult task in Hong Kong, yet some of the respondents shared that they were assumed to be the primary caregivers for their children in their families and they were completely consumed by the responsibility and daily demands. Their challenges were further topped by the misunderstanding and stigmas from their family members and the society, and this made them particularly lonely and burnt out. Research in Hong Kong has reveal that parents who have their children being diagnosed with ASD can feel helpless, shocked, confused and lonely, some are too ashamed even to disclose the diagnosis to their family members, and some nternalize the stigma and become upset with their affiliations with their children (27, 34). In our earlier paper, we also discussed the dynamics in families with children with neurodevelopmental disorders, while some family members were committed to offer support, some chose to distant themselves from the caring responsibilities (14). With this PII upholding self-care, they were offered the opportunities to reflect on their status, needs and directions, and made changes for sustainable caregiving and personal development.

Research has supported that self-compassion is essential for healthy caregiving (44). For caregivers who embrace self-compassion, they are more likely to avoid enmeshment and enjoy higher autonomy while building closer and satisfying relationships. Amid hardship, self-compassion also offers them anchors and self-soothing.

With many PIIs aim to teach children self-care skills, fewer PIIs are designed to teach caregivers the self-care that adults need (45, 46). As there has been a call for the clinical professionals and academics to attend to the self-care needs of caregivers (19), this study responds by documenting how the learning and practicing of self-care transform the lives of caregivers. With an emphasis on self-care, this study highlights the needs to consider the mental and physical health of caregivers in PIIs and to include self-care as an intervention component and an outcome indicator. When PIIs emphasize the interventionist role of caregivers and tend to monitor their performance, this research serves as one of the pioneers to advocate for the wellbeing of caregivers. As healthcare professionals and researchers are becoming more aware of the burdens of caregivers and the necessity for cultivating resilience in them, PIIs are believed to be a strategic tool to serve both the caregivers and the children with special needs (47).

Another breakthrough shared by the caregivers was changing the home setup. In the PII, the caregivers learnt that the seating arrangement at home would affect the parent-child interactions. When they were at the eye-level of their children, the young would have more opportunities to pay attention to the adults’ facial expressions, and this promoted parent-child engagement thus the child’s learning. The caregivers also learnt to observe the comfort level of both parties to sustain the joint engagement. They reported that after rearranging their home setup, the children became more focused, engaged and enjoyed the parent-child time more. The respondents also noticed that setting a dedicated space for parent-child interactions served as an environmental cue to remind them of the playtime, and this helped build their play routine. This cue was so effective that when some children saw their caregivers approaching the small table for playtime, they would proactively come to join in. While research have documented that environmental arrangement is a common strategy taught in PIIs for promoting the child’s willingness and likelihood to communicate, these studies have not yet explored its effects from the parents’ perspectives (48, 49). This research thus fills this gap by showcasing that from the caregivers’ experiences, the home setup not only facilitates communication, but it also serves as a visual reminder for building play routines.

The effectiveness of using environmental cues to shape and maintain health-related behaviors such as healthy lifestyle, eating habits and smoking habits have been demonstrated in previous research (50–52). The researchers find that when the environment is set up in ways that promote the desirable behaviors, the visual cues can propel people to choose what is hinted, hence people need to rely less on their will power and self-regulation to restrain themselves from doing what is undesirable. In this study, our novelty lies in offering evidence to show that environmental cues also works for building caregiver-child play routines thus relationships.

Discernment of the window for intervention was a breakthrough mentioned and cherished most by the caregiver-respondents. In the course, the caregivers were taught to observe the behaviors, emotions, interests and preferences of their children, and responded to them with a child-centered approach. Echoing other studies (6, 18, 19)., the feedback from trainers were found to be conducive for caregivers’ learning With the trainers providing feedback to the caregivers’ videos of playtime with their children, the caregivers learnt to observe the effectiveness of their existing parenting approaches and gradually adjusted their pace to align with their children’s. They realized that the best teaching approach was not to inculcate ideas into their children’s mind for this might overwhelm them. Instead, they needed to identify their children’s current language and cognitive capabilities and teach in ways that matched their current levels. Given that the feedback from trainers on actual practice has been found useful across studies (6, 18, 19), we encourage PIIs to include home visitations so that the trainers can offer practical support in environmental setup and in parent-child interactions.

Caregivers also learnt to follow into the children’s world by observing and mirroring the children’s play methods. They needed not find myriads play methods to teach different skills, but they could add little elements into the same activity to expose children to new learning. The caregivers felt more relaxed when they knew when to intervene and when to take a break. Shared with other studies that examine the effectiveness of the skills of imitation and mirroring (53, 54), this study reaffirms the values of synchrony in promoting parent-child interactions.

Through practicing this child-centered parenting approach, the caregivers found themselves having more empathy towards their children, and they developed more understanding about the children’s behaviors and emotions. When empathy was mentioned in the PII literature, it was usually in the context of the empathetic approach to engage caregivers or the cultivation of empathy in children (18, 55, 56). This study documents the growing of empathy in caregivers towards their children and how this affects the caregivers’ parenting.

This study offers unique values in understanding the critical transformation of caregivers in PII. Through examining the three breakthroughs, we highlight that the condition of caregivers—their wellbeing, capacities, concerns, needs, struggles, values and habits—play an important role in shaping their learning and applications of skills. Hence, we underscore the importance of recognizing where the caregivers are at and call for a more client-centered approach to PIIs.

This research, however, is not without limitations. We are aware that the sample size used in this research was rather small which contained only 21 caregiver-participants. Eighteen of them were mothers, hence the voices of fathers and other caregivers were underrepresented. This sample was drawn purely from an Asian community and the lived experiences were inevitably influenced by the local cultures. These characteristics of the sample inevitably constrain the generalizability of the findings. More qualitative research is to be conducted to explore the lived experiences of other caregiver groups in other settings.

In our data collection sessions, we heard some caregivers sharing how they applied the skills learnt in the localized WHO-CST on their children with typical development. This points to the possibility that the skills in PIIs can have a wider utility and greater impacts beyond its original target population. This can possibly be a future research direction.

## 5 Conclusions

This study serves to provide insights into the theory of change of PII by exploring the transformation processes of caregivers in a PII. Through focusing on their breakthroughs, it revealed their inner struggles and documented the key changes experienced and cherished by the caregivers. By zooming into the lived experiences of caregivers, it underscores the needs of considering the conditions, wellbeing and experiences of caregivers in PIIs for these factors directly affected the effectiveness and sustainability of the interventions. This study also encourages clinical professionals to include self-care techniques for caregivers as a core component in PIIs to strengthen their capacities and promote their wellbeing.

## Data Availability

The datasets presented in this article are not readily available because of the confidentiality agreement between the researchers and the participants but are available from the corresponding author on reasonable request. Requests to access the datasets should be directed to Dr. Paul Wong, paulw@hku.hk.
